# Stem Cell Extracellular Vesicles: Extended Messages of Regeneration

**DOI:** 10.1146/annurev-pharmtox-061616-030146

**Published:** 2016-10-28

**Authors:** Milad Riazifar, Egest J. Pone, Jan Lötvall, Weian Zhao

**Affiliations:** 1 Department of Pharmaceutical Sciences, University of California, Irvine, California 92697; 2 Sue and Bill Gross Stem Cell Research Center, University of California, Irvine, California 92697; 3 Chao Family Comprehensive Cancer Center, University of California, Irvine, Orange, California 92868; 4 Edwards Lifesciences Center for Advanced Cardiovascular Technology, University of California, Irvine, California 92697; 5 Department of Biomedical Engineering, University of California, Irvine, California 92697; 6 Department of Biological Chemistry, University of California, Irvine, California 92697; 7 Krefting Research Centre, Institute of Medicine, The Sahlgrenska Academy, Göteborg University, SE-405 30 Göteborg, Sweden; 8 Codiak BioSciences Inc., Woburn, Massachusetts 01801

**Keywords:** drug delivery, bioengineering, extracellular vesicles, exosomes, microvesicles, stem cells

## Abstract

Stem cells are critical to maintaining steady-state organ homeostasis and regenerating injured tissues. Recent intriguing reports implicate extracellular vesicles (EVs) as carriers for the distribution of morphogens and growth and differentiation factors from tissue parenchymal cells to stem cells, and conversely, stem cell–derived EVs carrying certain proteins and nucleic acids can support healing of injured tissues. We describe approaches to make use of engineered EVs as technology platforms in therapeutics and diagnostics in the context of stem cells. For some regenerative therapies, natural and engineered EVs from stem cells may be superior to single-molecule drugs, biologics, whole cells, and synthetic liposome or nanoparticle formulations because of the ease of bioengineering with multiple factors while retaining superior biocompatibility and biostability and posing fewer risks for abnormal differentiation or neoplastic transformation. Finally, we provide an overview of current challenges and future directions of EVs as potential therapeutic alternatives to cells for clinical applications.

## INTRODUCTION

Stem cells play vital roles in maintaining cellular homeostasis and restoring it upon tissue injury. During both embryonic development and regeneration of adult tissues, stem and progenitor cells must reach specific target cells that could be either in close proximity or at a distance. Whereas embryonic development typically occurs in a controlled environment with minimal external disturbances, adult stem cells face frequently the additional challenge of operating in a harsher environment induced by trauma or infection with associated pathological and inflammatory responses. Stem cells are able to communicate with nearby and distant cells through soluble factors and direct cell-cell contact by long and thin tubular appendages such as cytonemes and cilia, as well as via detached extracellular vesicles (EVs). These vesicles possess properties of freely diffusing factors and the extensive membrane and cytoplasmic organization of cells. They range from approximately 30 nm to 3,000 nm in diameter and have a distinct biomolecular composition and, therefore, functions that depend on EV subtype, cell source, and conditions (see the sidebar titled Definitions and below).

DEFINITIONS

Apoptotic bodies or blebs (ABs): relatively large (up to several microns) cell fragments released from cells undergoing apoptosis
Exosomes: biological vesicles approximately 30–200 nm in diameter that originate as ILVs in MVBs (see below) and become released as EVs upon MVB fusion with the plasma membrane
Extracellular vesicles (EVs): all-encompassing term for cell-derived small vesicles; includes exosomes, microvesicles, and ABs
ILVs: intraluminal vesicles derived from invaginations of early endosomes to be contained within MVBs; referred to as exosomes upon their release outside the cell
Liposomes: synthetic, nanometer- to micrometer-sized lipid vesicles; frequently engineered to carry and release cargo in a targeted and controlled manner
Microvesicles/ectosomes: biological vesicles approximately 50–300 nm in diameter that bud from the plasma membrane
MVBs: multivesicular bodies derived from endosomes that invaginate inward to form ILVs
Nanoparticles: synthetic, nanometer-scale particles made of polymers, metals, or other materials; frequently carry and release cargo in a targeted and controlled manner

EVs were initially considered cellular debris or a way to excrete unneeded or toxic products from cells, but their ancient evolutionary origins and their conserved mechanisms of generation indicate that EVs perform essential physiological roles in cell-cell communication (1, 2). EVs are found in all bodily fluids examined thus far, and most cells can secrete EVs to varying degrees: Some cell types release EVs under steady-state conditions, whereas other cell types, particularly immune and stem cells, can be induced by various stimuli, including stressors and physiological mediators, to release EVs (1–4). Cells can internalize EVs through several mechanisms, including direct membrane fusion, clathrin-dependent endocytosis, caveolin-mediated uptake, macropinocytosis, and phagocytosis mediated by specific receptors (5). EVs are thought to play roles in establishing and maintaining cell and tissue polarity, including as vehicles for morphogen and nucleic acid release and distribution during embryonic development and adult tissue regeneration (6, 7). For example, during embryonic development, lipidated signaling ligands for the Wnt and Hedgehog (Hh) pathways not only flow across cells along plasma membranes but have also been found on the surface of EVs, thereby contributing to the actions of EVs in stem cell activation for cellular homeostasis and tissue repair after injury (7, 8). In addition, in adult injured tissues, EVs loaded with apoptotic and other signals can be received by stem cells to help direct their healing response. In turn, stem cell EVs contain a wide variety of lipids, proteins, and nucleic acids that can help heal distressed parenchymal cells (9, 10).

Thus, the study of the roles of endogenous stem cell EVs, although still in its initial stages, has provided key background and rationale on using exogenous and bioengineered stem cell EVs to help treat injured or diseased tissues. Furthermore, in the context of cell therapy, exogenous cells infused intravenously (IV) are normally trapped in the lungs (the pulmonary first-pass effect) and also in other filter organs such as the liver, spleen, and kidneys (11, 12). There, the trapped cells disintegrate into smaller vesicles, including apoptotic bodies (ABs) and likely other types of EVs, which may traffic to distant organs to exert therapeutic and biological functions (12, 13). Therefore, investigation of stem cell EVs will also shed light on the therapeutic functions of stem cells following their systemic infusion or local transplantation (11, 14–16).

EVs fall in the intermediate range of current biologics for tissue regeneration and immunomodulation (i.e., between larger entities, such as platelets, apoptotic cells, whole stem cells, and immune cells, and biopharmaceuticals, such as small-molecule drugs, peptides, growth factors, antibodies, and nucleic acids). As EVs are loaded differentially and combinatorially with multiple types of proteins together with coding and regulatory nucleic acids, the responses they induce may be more complex and longer-lasting compared to those elicited by individual biomolecules (2, 9, 10, 17). In addition, EVs can serve as extracellular stores that release molecules with a different spatial and temporal distribution profile compared to whole cells. An example is during immune responses to influenza infection, whereby neutrophils deposit EVs on collagen tracks to release the chemokine CXCL12 slowly and thereby attract anti-influenza cytotoxic T lymphocytes (18).

One of the most interesting and emerging functions of EVs may be in the maintenance of tissue homeostasis, as EVs released from injured tissue have been found to influence stem cells, and conversely, stem cell–derived EVs have been found to support injured tissue. This role of EVs in the cross-talk between stem cells and parenchymal cells, and how this can be exploited therapeutically, is the main subject of this review; we refer readers to several recent reviews on additional aspects of EVs (1, 2), their bioengineering (19), and their therapeutic applications (20). We also review how stem cell EVs can be engineered for therapeutic or diagnostic applications in ways analogous to those pioneered by research on nanoparticles and liposomes (21, 22). We conclude with a discussion on issues related to the clinical translation of EVs as therapeutics.

## EV COMPOSITION, DIVERSITY, AND FUNCTIONS

**Figure 1:**
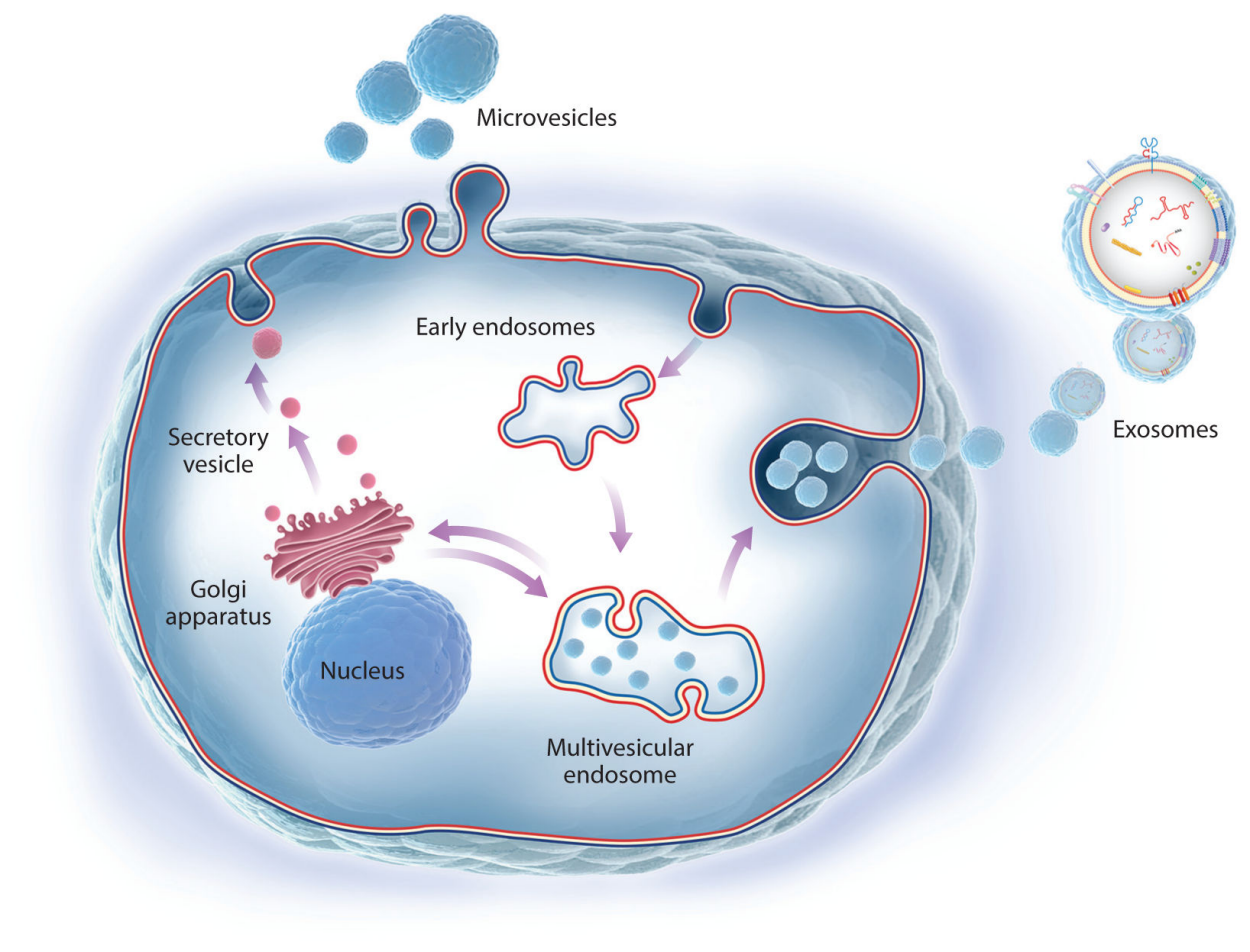
Biogenesis of extracellular vesicles (EVs). Microvesicles derive from the plasma membrane in a way reminiscent of the reverse of endocytosis. Exosomes are generated as intraluminal vesicles (ILVs) inside multivesicular bodies (MVBs), which, in turn, originate either from invaginations of the plasma membrane or from intracellular organelle membranes. Exosomes are created during two successive membrane invaginations: Membranes invaginate inward to generate MVBs, which in turn invaginate inward to generate ILVs.

Endocytosis, exocytosis, and EV biogenesis are functions that have evolved as ways in which cells maintain surveillance of, and in turn influence, the surrounding environment and neighboring cells, although researchers are only beginning to understand the stimuli or driving forces for EV release (1, 2). EVs are generally divided into three main types: exosomes emitted from intracellular endosomes, microvesicles that bud directly from the plasma membrane, and ABs released during apoptosis (see the sidebar titled Definitions and Figure 1). The nomenclature of different EV preparations reported in the literature has been inconsistent partly because of a lack of uniform operational definitions (e.g., centrifuged ABs pellet at about 2,000 g, microvesicles pellet at about 16,000 g, and exosomes pellet at 100,000 g), although scientific societies such as the International Society for Extracellular Vesicles (ISEV) (23) and others are establishing guidelines on EV research.

### Types and Functions of EVs

**Figure 2:**
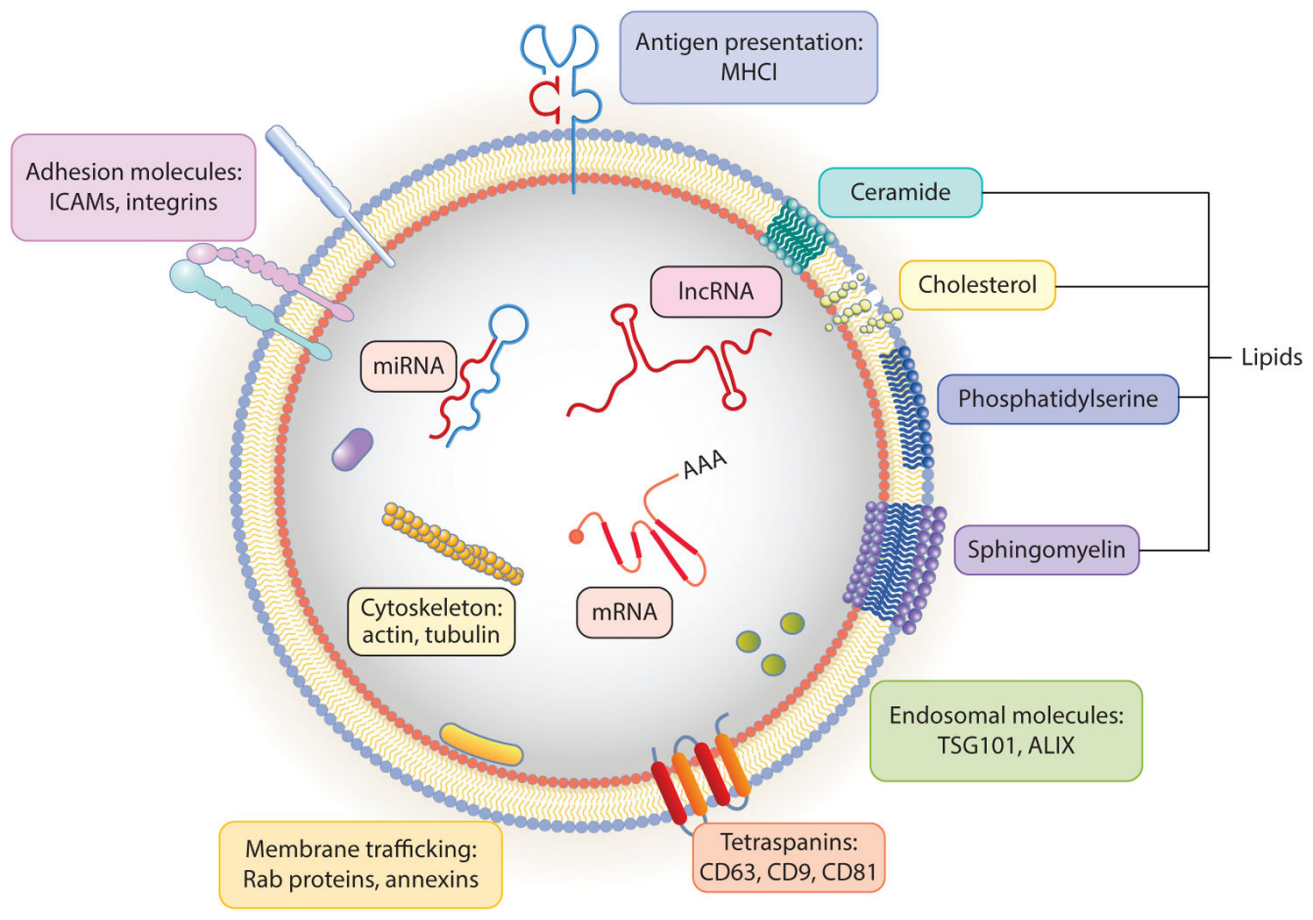
Anatomy of EVs. EVs contain several characteristic lipids, proteins, and RNA molecules depicted here schematically. Because exosomes are generated when ILVs are released upon MVB fusion with the plasma membrane, exosomes have the same membrane leaflet composition as ILVs. Surface proteins include MHC molecules, ICAMs, integrins, tetraspanins (e.g., CD63, CD81), TSG101, and ALIX. Lumen proteins include cytoskeletal actin and tubulin, Rab GTPases, and inner membrane leaflet-associated proteins. RNAs include mRNA, lncRNA, miRNA, piRNA, vaultRNA, Y-RNA, rRNA, and tRNA (not all depicted here). During MVB biogenesis and development, ILVs may incorporate additional lipids, proteins, and nucleic acids in a nonspecific bystander way or by specific recruitment. Abbreviations: EV, extracellular vesicle; ICAM, intercellular adhesion molecule; ILV, intraluminal vesicle; lncRNA, long noncoding RNA; MHCI, major histocompatibility complex class I; miRNA, microRNA; mRNA, messenger RNA; MVB, multivesicular body; piRNA, picoRNA; rRNA, ribosomal RNA; tRNA, transfer RNA.

Exosomes were first examined in detail during reticulocyte maturation to red blood cells, wherein the transferrin receptor is lost gradually by incorporation in the membranes of intraluminal vesicles (ILVs) inside multivesicular bodies (MVBs) that are eventually released from the cell as free exosomes (24). MVBs originate from invaginations of the plasma membrane; from intracellular organelle membranes; or from membranes nucleated de novo, similar to autophagic membranes, but which rely on multisubunit endosomal sorting complexes required for transport (ESCRT). When some of these critical molecules for exosome biogenesis are inhibited, exosome secretion is generally reduced, although because other processes such as cell division and additional vesicular trafficking events are also affected, the importance of exosomes in homeostasis and tissue repair remains to be fully elucidated (1, 2). Use of genetic approaches, such as cre-lox reporter techniques (25, 26), will likely clarify these questions. Exosomes are enriched for specific biomolecules, including lipids important for structural stability, tetraspanins important for curvature generation and membrane protein organization and stability (27), pentaspan membrane glycoproteins (28), and other cell-specific proteins, as well as several types of nucleic acids (Figure 2). Selected examples of proteins and nucleic acids found in EVs from stem and parenchymal cells that are responsible for therapeutic efficacy or other phenotypic changes in recipient cells are listed in Table 1.

Microvesicles, sometimes also called ectosomes, originate from outward invaginations of plasma membrane regions in a manner roughly reminiscent of the reverse of endocytosis. Microvesicles contain plasma membrane proteins as well as cytosolic proteins, nucleic acids, and other metabolites. Because microvesicles originate by plasma membrane pinching, they are exposed continuously to cytoplasmic material, unlike ILVs, which are encased within MVBs. Nevertheless, active targeting or sorting mechanisms can enrich microvesicles with nucleic acid, protein, and lipid constituents, and, akin to exosomes, the biogenesis of microvesicles could also use ESCRT to complete vesicle budding (29).

ABs result from fragmentation of apoptotic cells and therefore are composed of plasma and organellar membranes and partially hydrolyzed nuclear and cytoplasmic material. ABs play key roles in cellular homeostasis, including induction of immunogenic tolerance in the absence of infection, which is used in animal studies and clinical trials (30–32). Some ABs are likely released when IV-infused stem cells are trapped in filter organs and may influence the therapeutic outcome.

### Lipid, Protein, and Nucleic Acid Composition of EVs

As mentioned for reticulocyte exosomes, alteration of membrane lipid and protein composition is one important function of EVs (33). The lipid profile in EV subsets depends on the cell type (2), membrane origin, and the activity of membrane lipid scramblases, flippases, or floppases. There are few studies on the lipid distribution in different membranes (including lipid rafts) of stem cells; nevertheless, the presence of certain membrane proteins that bind to specific lipids, such as lactadherin and annexins (which bind to phosphatidylserine) and prominins (which bind to cholesterol), has been reported on stem cell EVs (34) (Figure 2). This phenomenon may reflect a distinct lipid distribution in stem cell EVs compared to the average distribution in their originating stem cells or that of EVs from other cells.

EVs contain integral membrane proteins such as tetraspanins and pentaspan proteins, peripheral membrane proteins such as lactadherin and annexins, submembrane actin and intermediate filaments, and intravesicular proteins that are either soluble or associated with the above proteins (Figure 2). Interestingly, prominin-1 (CD133) and prominin-2, which associate with cholesterol, are highly enriched in stem cell membrane projections, cytonemes, cilia, and microvilli, as well as on EVs, although the mechanisms by which prominins contributes to stemness, sensing, differentiation, or other stem cell functions remain unclear (34–36). Tetraspanins play particularly prominent roles in cytonemes and EVs by giving them curvature and strength and by regulating the spacing, distribution, trafficking, and fusion of membrane proteins and their interacting partners (37). This naturally organized and interlaced membrane texture likely accounts for EVs being nearly as hard as viruses and about an order of magnitude harder than synthetic liposomes, inferred by their high elastic modulus and ability to deform elastically while maintaining vesicle integrity as measured by atomic force microscopy (38). Indeed, their intrinsic durability and natural biocompatibility may render EVs particularly suitable as delivery vehicles for natural and synthetic therapeutics.

EVs contain a variety of nucleic acids (Figure 2). Circulating cell-free nucleic acids might be found largely within EVs or associated in various lipoprotein and riboprotein particles, as the half-life of naked nucleic acids in serum is low (2, 39, 40). Following the landmark discovery that functional mRNA can be transferred through exosomes (41), numerous studies have shown biological roles of EVs shuttling RNAs in cell-cell communication (39) (Table 1). Nucleic acids found in EVs include virtually all RNA species [messenger RNA (mRNA), ribosomal RNA (rRNA), and transfer RNA (tRNA) required for translation and long noncoding RNA (lncRNA), microRNA (miRNA), picoRNA (piRNA), vaultRNA, and Y-RNA involved in regulation of various other aspects of gene expression] (2). In particular, lncRNAs are known to bind to complementary DNA or RNA targets, but also to act as aptameric ligands for other biomolecules such as proteins, to regulate gene transcription, posttranscriptional, and epigenetic events, especially during developmental and differentiation processes (42). Importantly, miRNAs, a distinct class of small (approximately 22 nucleotides), single-stranded, noncoding RNAs, play critical functions in the regulation of cellular gene expression by binding to complementary sequences in the target mRNAs, leading to either translational repression or target degradation of the specific mRNAs (43). Interestingly, miRNAs transferred via EVs to other cells can alter recipient cell responses, as miRNAs act in catalytic-like fashion and can target multiple critical nodes in intracellular pathways (2, 39, 44). Illustrative examples of EVs transporting stem cell and parenchymal mRNA, miRNA, and lncRNA are discussed in subsequent sections. Nucleic acids and proteins of EVs have been curated in several databases, including ExoCarta (http://www.exocarta.org) and EVpedia (http://www.evpedia.info).

### EVs in the Spreading of Intercellular Signaling

In contrast to intracellular signaling, which occurs by membrane components, soluble protein factors, and scaffolded complexes (45), multicomponent extracellular signaling occurs rarely, with notable examples including blood clotting complexes, amyloid complexes and other aggregates, and complexes of antigens with complement factors or antibodies. The fact that EVs host multiple proteins endows them with greater capacity for information content compared to traditional single-molecule messengers such as hormones, growth factors, cytokines, and other mediators (17, 32). And because EVs contain membrane receptors that are organized as in the source cell, they may convey similar responses in recipient cells. For example, exosomes derived from antigen presenting cells bear major histocompatibility complex class II (MHCII)-peptide complexes (and presumably other adhesion and costimulatory receptors in their proper stoichiometry and spacing), which can activate cognate T cells (32).

Signal transduction at the plasma membrane also initiates negative feedback loops to extinguish the signal, including by endocytosis of receptor-ligand complexes, thereby helping to distinguish persistent from spurious signals. Although endocytosis was initially thought to dampen signaling by degrading active receptor-ligand pairs, it is required for some signaling events emanating from intracellular endosomes (46). Thus, depending on the fate of the early endocytic vesicles or MVBs, their fusion with the plasma membrane to emit exosomes may spread intracellular endocytic signaling (47) to other target cells (48) (Table 1), whereas the fusion of exosomes with lysosomes or autophagosomes can extinguish signaling. In fact, signal-inducing EVs have also been variously referred to as intercellular signaling devices (49), circulating signaling modules (50), intercellular signalosomes (3), and intercellular signaling organelles (51, 52). Similarly, EV-mediated signal propagation across cells has been termed exosomal targeted receptor activation signaling (53) or intercellular transfer signaling (48). This EV-mediated intercellular communication is expected to be particularly applicable to motile heterogeneous cells (in which communication through gap junctions is not as feasible as in stationary tissues), such as immune cells or stem cells mobilized from the bone marrow, fat deposits, or other stores. Because tissue-resident and exogenous IV-infused stem cells are typically rare and not necessarily in direct contact with the injured cells, they could utilize soluble factors as well as EVs and their contents to reach the layers of injured cells or coordinate tissue-wide responses.

## ENDOGENOUS EVS IN EMBRYONIC DEVELOPMENT AND ADULT TISSUE HOMEOSTASIS

### EVs and Morphogen Distribution in Development

**Figure 3:**
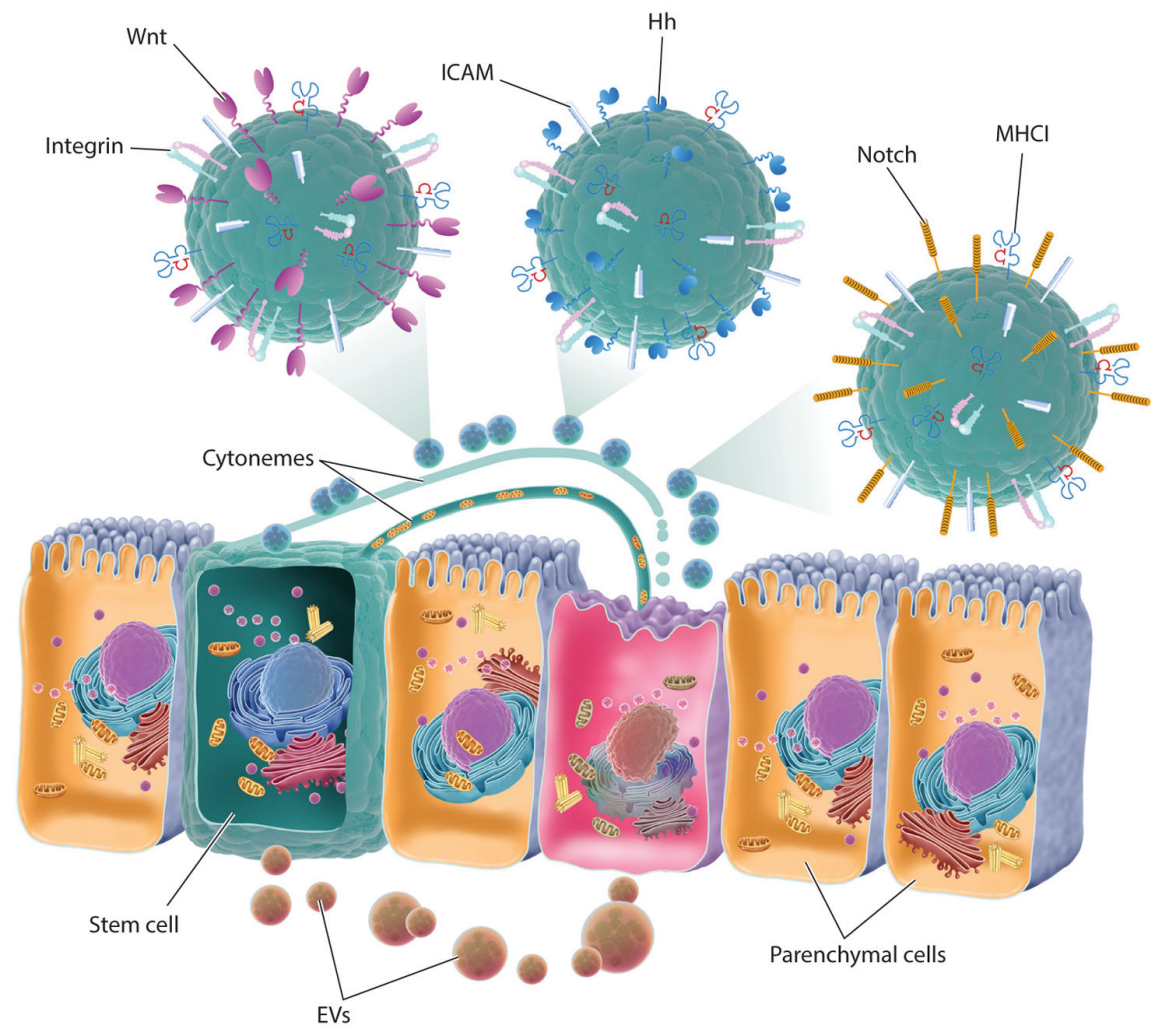
EVs in bidirectional communication between stem and parenchymal cells. Stem cells may sense parenchymal cell injury or distress by receiving parenchymal EVs, and in turn, stem cell EVs harboring prohealing RNAs and proteins may be received by parenchymal cells, maintaining tissue homeostasis. Cellular cytonemes and cilia can establish direct contact between cells for transfer of biomolecules and can also serve as transmission points for EVs. During development, cytonemes and EVs also carry morphogens such as Wnt, Hh, and Notch ligands. Abbreviations: EV, extracellular vesicle; ICAM, intercellular adhesion molecule; MHCI, major histocompatibility complex class I.

The development and growth of the embryo during development, and the actions of adult stem cells during tissue regeneration, are controlled by signals that have various concentration gradients and topologies (soluble, particulate, or membrane-, cytoneme-, or vesicle-bound). Soluble growth and differentiation factors, referred to frequently as morphogens, may be distributed along gradients from their source and captured on extracellular matrix or membrane proteoglycans before engaging their cognate receptors to initiate signaling. Although stem cell differentiation factors, such as Wnt and Hh, are known to be lipidated small proteins flowing along membranes from parenchymal cells or nearby niche and stromal cells to recipient stem cells (54), recent reports indicate they may also be emitted in other forms, including on EVs (7, 8, 52, 55) (Figure 3). Furthermore, morphogens may be secreted on EVs by various invertebrate and vertebrate cell types (8, 56) and in some cases secreted in a polarized way in extracellular compartments such as immune and nerve synapses, epithelial compartments, and stem cell niches (55). For example, intestinal epithelial cells secrete two types of exosomes (57) from their apical and basolateral membranes enriched in distinct phospholipids and proteins (6, 58).

Thin cell projections that have been termed filopodia, dendrites, tunneling nanotubes, or cytonemes transport morphogens and other signals during embryonic development (59). Cytonemes lack the typical 9 + 2 microtubules found in cilia but instead are organized by a variety of membrane proteins, particularly tetraspanins and certain lipids, to serve as long-ranging platforms of cargo distribution, including on EVs, from source to recipient cells (37, 60). During *Drosophila* embryonic development, cytonemes from wing cells orient toward source cells that produce morphogens such as Wnt, Hh, epidermal growth factor, fibroblast growth factor (FGF), and decapentaplegic; this polarized orientation and cytoneme length are typically reduced when the ligands are distributed uniformly under experimental conditions (59, 61). Similar roles for cytonemes in morphogen transport and signaling have been described in vertebrates (62), in which Hh signaling occurs only in cilia. Stem cells may use cytonemes to reach distant tissue cells—for example, to transfer cytoplasmic content, including mitochondria, from mesenchymal stem cells (MSCs) to cardiomyocytes (63) (Figure 3). Another possibility by which mobilized marrow or resident stem cells may reach injured cells is via telocytes, which are cells with a prominent primary cilium that researchers propose is involved in sensing parenchymal health and distress signals and conveying them to stem cells (64). Platelets activated under shear flow also form cytoneme-like appendages termed flow-induced protrusions (FLIPRs), which pinch off microvesicles at their tips (65) and are reminiscent of neutrophil extracellular traps (NETs) reported to play roles in immune and autoimmune responses and thrombosis (66). A variety of other spherical and tubular EVs (possibly from severed cytonemes) have been reported in plasma (67).

Cytonemes and cilia may serve as terminal transmission points for EVs, possibly in the polarized transfer to certain compartments such as neural and immune synapses, endocrine sites, and blood (55, 59, 68). Thus, in *Caenorhabditis elegans*, ciliated sensory neurons release microvesicles (but not MVB-derived exosomes) that function in mating behavior (69). During the development of the neuromuscular synapses in *Drosophila*, Wnt and Wnt-binding proteins reemitted and received across the synapse on exosomes (70, 71). In the developing wing epithelium of *Drosophila*, Wnt and Hh are transported on lipoprotein particles (72) as well as on bona fide exosomes (72, 73), although the Wnt gradient may form independently of exosomes (74). Similar to Wnt, the morphogen Hh was found in exosomes budding from apical microvilli of the mouse ventral node during embryonic development (75). Another study reported that EVs bearing Hh could induce effective Hh signaling in recipient cells only when EVs also carried integrins (76), possibly pointing to the importance of adhesion and ligand avidity for EV activity.

Secretion of exosomes containing Hh-related peptides during cuticle development in *C. elegans* requires acidification of the endosomes by vacuolar ATPases (V-ATPases) (77); a similar requirement for an acidifying V-ATPase has been made for Wnt signaling (78, 79). This acidification of Wnt and Hh ILVs in MVBs may not necessarily serve to hydrolyze contents as it does in digestive lysosomes, phagosomes, and autophagosomes; instead, it could potentially serve to strip the ligand off the receptor (47) or to facilitate recognition of pH-dependent conformations of specific Wnt and Hh factors by their internalizing or other intracellular receptors. The Notch signaling ligand Delta-like 4 can also be distributed in exosomes, in which form it may regulate long-distance angiogenesis during tissue regeneration (80, 81). Akin to Wnt and Hh signaling, Notch signal transduction involves receptor-ligand internalization in ILVs within MVBs (82), although it is not clear if these Notch-bearing ILVs are processed further or released from MVBs as exosomes for other rounds of intercellular signaling. Overall, these reports point to a likely role for EVs in spreading signal transduction through multiple parenchymal or niche cells to activate and differentiate stem cells.

### EVs in Injured Adult Tissues

Apoptosis and its end products, ABs, play a critical role during embryonic development (e.g., in regulation of limb webbing) and during adult cellular homeostasis by controlling the process of apoptosis-induced proliferation (83) and regulating immune responses (84). ABs contain several molecules that are normally absent on the outer membrane leaflet of healthy cells and serve as find-me and eat-me tags (such as the lipids phosphatidylserine and lysophosphatidylcholine and the proteins calreticulin, lactadherin, and annexins) to recruit and activate phagocytes (84). ABs may be resorbed through efferocytosis not only by professional phagocytes (e.g., macrophages) but also by nonprofessional phagocytes such as neighboring parenchymal cells (85), underlying progenitor cells, fibroblasts, and MSCs (86). One hypothesis is that early progenitor cells may uptake EVs or even AB remnants of the deceased cells loaded with nucleic acids, lineage-determining transcription factors, or other signals that may then contribute to terminal differentiation of the progenitor cell. Indeed, several recent reports, particularly from the laboratories of Quesenberry and Camussi, indicate that EVs released from parenchymal cells influence underlying stem cells, and conversely, EVs released from stem cells influence parenchymal cells (9, 10, 87) (Figure 3). Thus, parenchymal lung or prostate cell microvesicles deliver their mRNA cargo into bone marrow cells, thereby exerting tissue-specific changes in these cells (88–90). These stable changes in recipient stem and progenitor cells may depend on their cell cycle and on the extent of injury in parenchymal cells emitting EVs (91–93). Conversely, because mobilized bone marrow MSCs or tissue-resident stem cells, which are sparsely distributed (94), may not be able to reach all the injured parenchymal cells directly, they might use cytonemes and EVs to deliver prohealing factors (such as certain lipids, signaling receptors or ligands, enzymes, lineage-determining transcription factors, and coding and regulatory RNA) that target pathways in recipient cells to restore cellular and tissue homeostasis (Table 1).

## STEM CELL EVS AS THERAPEUTICS: A NEW PARADIGM FOR CELL-FREE THERAPY

Ex vivo–manufactured natural and engineered stem cell EVs administered to a patient can plausibly be used to recapitulate some or most of the therapeutic benefits of whole stem cells while potentially avoiding pitfalls associated with the latter, such as neoplastic transformation (95) and immune response activation (96). Because of their acellular status and small size, EVs present the following advantages compared to whole cells: They may avoid entrapment in filter organs and potentially cross other biological barriers, they can be modified and loaded with reagents of interest, they can be stored without significant loss in activity, and they may have lower side effects and other risks associated with cell transformation and immunogenicity (2, 13). In addition, the fact that EVs host multiple types of biomolecules potentially allows them to target different therapeutic mechanisms simultaneously, which is not possible by traditional single-molecule entities. Furthermore, owing to their natural biogenesis machinery, EVs are amenable to genetic engineering approaches similar to those of whole cells for uploading cargo of interest, whereas this is as yet generally incompatible with nanoparticle and liposome technologies.

### Preparation and Engineering of Exogenous Stem Cell EVs

EVs are isolated based on differential ultracentrifugation (97), gel filtration on special matrices (98), or tangential flow filtration (TFF) (99), with the latter being suitable for industrial-scale production. Because exogenous stem cells infused IV in patients are subjected to a variety of stresses (e.g., hypoxia resulting from cellular aggregation, nutrient starvation, shear stress, and deformation of infused cells), it seems reasonable to stimulate stem cells during their expansion in cultures in vitro with various pro- or anti-inflammatory cytokines, growth factors, nutrients, oxygen concentrations, and so on to aid in the production of EVs with improved therapeutic roles. For instance, cytokines such as interferon-γ and tumor necrosis factor-α induce the expression of programmed death-ligand 1 and MHCII (which are critically involved in the orchestration and resolution of the inflammatory response) and therefore are used frequently to condition stem cells before harvesting their EVs (100).

**Figure 4:**
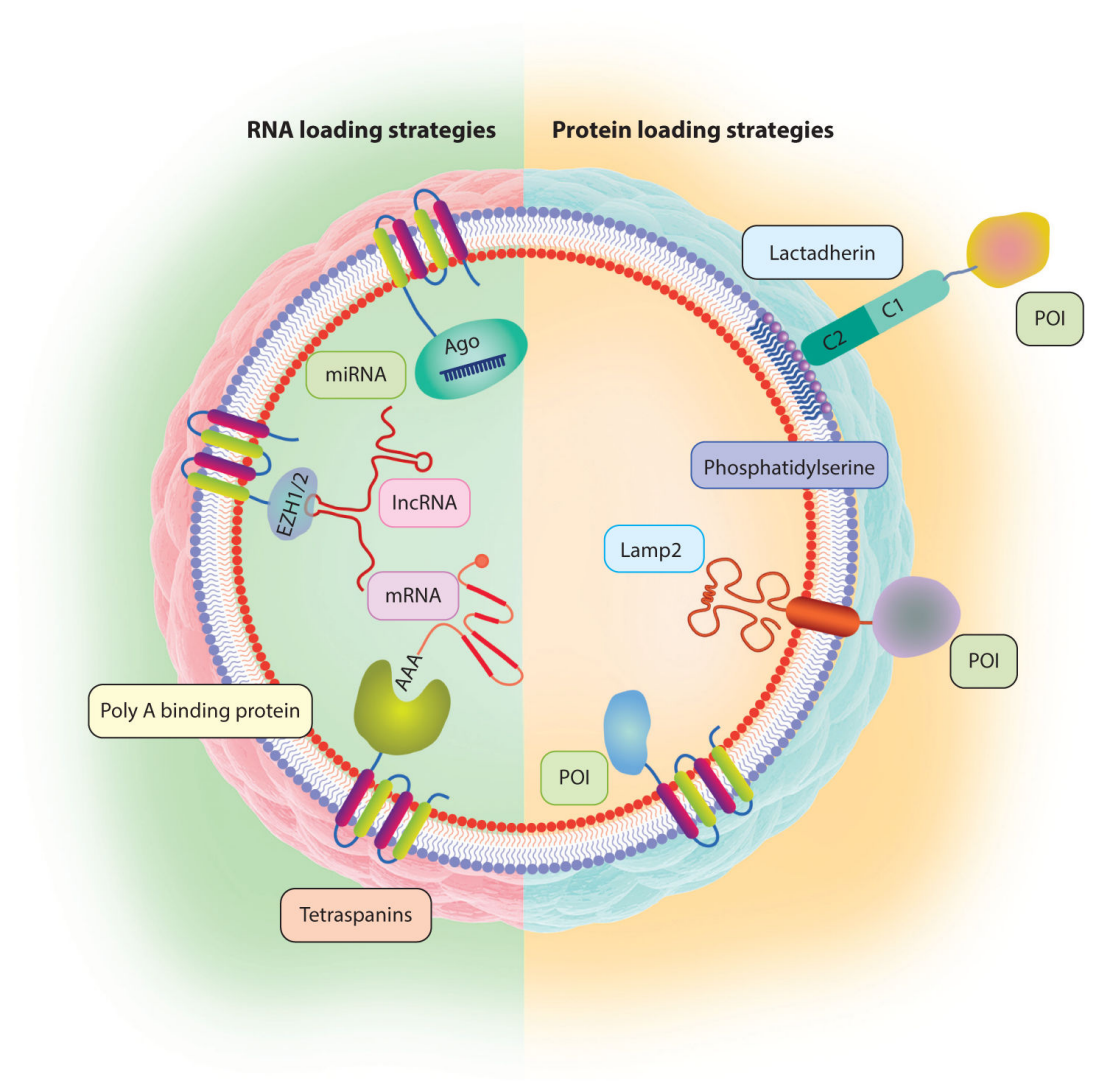
Bioengineering EVs for targeted therapy. EVs can be engineered to incorporate nucleic acids and POIs. To concentrate a protein in the lumen of EVs, its interacting partner can be fused with tetraspanin CD63. Likewise, miRNAs can be enriched into EVs by fusing Ago protein with CD63. Poly A binding protein, which binds mature mRNAs, can recruit mRNAs selectively into EVs. lncRNAs can be enriched in EVs by fusing motifs from polycomb repressive complex 2 (EZH1 and EZH2) with tetraspanins. To incorporate POIs on the membranes of EVs, sequences coding for acylation motifs or membrane-spanning helices can be fused to the POIs. Abbreviations: EV, extracellular vesicle; lncRNA, long noncoding RNA; miRNA, microRNA; POI, protein of interest.

The three main types of EV cargo are proteins, RNAs, and small-molecule drugs, which can be loaded into EVs by active approaches (i.e., incorporation during EV biogenesis, such as by genetic modification of the cells) and passive approaches [i.e., incorporation after EV secretion, such as by electroporation (101) or chemical conjugation (102)]. Engineering of stem cell EVs typically takes advantage of their natural production processes and properties and further combines them with genetic or nongenetic designs to add functionalities (e.g., targeting, therapy, sensing, imaging) (Figure 4). Receptor-ligand pairs can be used to deliver modified EVs bearing receptors that bind to the ligands on target cells, therefore reducing off-target effects while increasing efficacy. For example, Epstein Barr virus–transformed B cells secrete exosomes that contain glycoprotein 350 and thus target other CD21^+^ B cells selectively (103), whereas neuron-specific rabies viral glycoprotein peptide—fused genetically to Lamp2 to enrich it on EVs—delivers a specific small interfering RNA (siRNA) to the brain (104). Genetic modification of cells with CD47 or CD200 may protect them against phagocytosis, whereas CD55 and CD59 don’t-eat-me tags provide protection from complement-mediated lysis of EVs (105, 106).

Tags that bind specific types of nucleic acids are particularly relevant to engineering stem cell EVs containing RNAs targeting a particular pathway or pathways. Poly A binding protein, which binds mature mRNAs, can be used to recruit mRNAs selectively into EVs. Alternatively, a zipcode-like, approximately 25-nucleotide sequence present in the 3′-untranslated region (3′UTR) of certain mRNAs can be incorporated into the 3′UTR of the mRNA of interest and be recruited by the Z-DNA binding protein 1 (ZBP1) (107). Likewise, to enrich EVs selectively with miRNAs, Ago protein or catalytically dead Dicer/Drosha can be fused [e.g., to the tetraspanin CD63 (both C and N termini are intravesicular)] to load miRNAs or pre-miRNAs and pri-miRNAs, respectively. Polycomb repressive complex 2 (gene symbols *EZH1* and *EZH2*) recognizes certain lncRNAs selectively and therefore can be used to enrich lncRNAs into EVs. Other RNA types, such as P-element induced wimpy testis (piwi RNA) can be loaded in EVs by recruiting them via motifs of the interacting proteins piwi-like RNA-mediated gene silencing 1 (gene symbol *PIWIL1*), piwi-like RNA-mediated gene silencing 4 (*PIWIL4*), or piwi-like RNA-mediated gene silencing 2 (*PIWIL2*) (108). In addition, vaultRNAs can be enriched into EVs by overexpressing major vault protein (gene symbol *MVP*) fused with CD63.

Loading of EVs is not limited to biological cargo but can also include small-molecule drugs, which are typically incorporated in EVs through postisolation methods such as electroporation, transient osmotic shock, or reversible chemical covalent modification. For example, dendritic cell (DC)-derived EVs loaded with doxorubicin delivered it to breast cancer cells and inhibited tumor growth without major toxicity (109). Similarly, curcumin (a naturally occurring anti-inflammatory compound) was encapsulated inside EVs and delivered to the brain to reduce inflammation (110).

### Exogenous Stem Cell EVs in Tissue Regeneration and Immunomodulation

Most studies of stem cell EVs have involved embryonic stem cells (ESCs), hematopoietic stem or progenitor cells, neural stem cells, endothelial progenitor cells (EPCs), and MSCs (13, 20, 111–114). Human ESC–derived microvesicles induced dedifferentiation and pluripotency in their target retinal Müller cells by selectively transferring mRNA (Oct4 and Sox2), miRNA (290 cluster), and proteins that altered gene expression and epigenetic state, raising the possibility of employing EVs to enhance the retina’s regenerative capacity (115). Murine ESC–derived exosomes enhanced the healing of infarcted hearts by increasing cardiomyocyte survival and neovascularization and reducing fibrosis (116). Similarly, microvesicles from human EPCs transferred mRNA and miRNA that activated endothelial cell proliferation to support revascularization of injured murine tissue (87, 117, 118). In addition, exosomes from human cardiac progenitor cells expanded ex vivo regenerated injured murine hearts by inhibiting apoptosis and increasing the proliferation of cardiomyocytes and endothelial cells (119).

Stem cells, such as ESCs and induced pluripotent stem cells (iPSCs), have tremendous promise, but at present they remain constrained for clinical use by safety issues that include their immunogenicity, incomplete differentiation, and neoplastic transformation (95, 120). In current practice, MSCs (usually obtained from adult bone marrow, adipose tissue, or neonatal umbilical cord) are among the most widely used types of adult stem cells in animal studies and human clinical trials (121, 122) for reasons that include easy isolation and expansion in culture, their comparatively infrequent abnormal differentiation or neoplastic transformation (123), their natural tropism to injured tissues (124) or cancer (123), and their potent immunomodulatory properties (122, 125, 126). For these reasons, MSC EVs are also among the most widely studied stem cell EVs and are reported to help heal a variety of stressed parenchymal tissues (113, 122), as discussed briefly below.

In the subtotal nephrectomy murine model of kidney regeneration, microvesicles from MSCs, akin to intact MSCs, protected against renal injury by decreasing the levels of creatinine, uric acid, lymphocyte infiltration, and fibrosis (127). Similarly, MSC-derived microvesicles improved the recovery from glycerol-induced acute kidney injury by shuttling mRNAs associated with the mesenchymal phenotype and inducing proliferation of renal tubular cells (128). In a murine model of fibrotic liver disease induced by carbon tetrachloride, exosomes derived from human umbilical cord MSCs reduced the surface fibrous capsules by reducing collagen deposition and alleviated hepatic inflammation by increasing the levels of transforming growth factor-β (129). In a rat skin burn model, MSC exosomes carrying Wnt4 enhanced cutaneous healing by increasing the expression of CK19, proliferating cell nuclear antigen, and collagen I, which cumulatively enhanced functional re-epithelization (130). In an acute lung injury murine model induced by lipopolysaccharides, MSC-derived microvesicles reduced pulmonary inflammation and enhanced tissue recovery, mediated in part by delivery of angiopoietin 1 and keratinocyte growth factor (FGF7) (131). Similarly, exosomes from bone marrow or umbilical cord MSCs were enriched for miR-16 and miR-21 and enhanced lung tissue recovery in a murine model of hypoxia-induced pulmonary hypertension by suppressing the production of monocyte chemoattractant protein-1 to reduce the influx of macrophages and by reducing the activation of signal transducer and activator of transcription 3 to inhibit vascular remodeling (132). MSC EVs enhanced recovery in a mouse model of myocardial ischemia-reperfusion injury by increasing the levels of ATP and NADH, increasing phosphorylated Akt and phosphorylated glycogen synthase kinase-3β, reducing phosphorylated mitogen-activated protein kinase 8, and decreasing oxidative stress (133). In the stroke model produced by cerebral artery occlusion in rats, MSC exosomes delivered miR-133b to neural cells to enhance neurite outgrowth after stroke (134) and promoted functional recovery by increasing the number of proliferating neuroblasts and endothelial cells (135). There is only one report to date of EVs administered under compassionate care to a patient suffering from graft versus host disease (GvHD); no adverse effects were noted (136). Clinical trials with additional patients are expected to commence in the near future. Taken together, these results suggest that MSC EVs mediate therapeutic effects in different disease models via multiple mechanistic pathways (Table 1) and may provide a novel approach for the treatment of degenerative, inflammatory, and acute injury–associated diseases (122).

### EVs in Cancer, Metastasis, and Drug Resistance

We discuss briefly the effects of EVs on cancer, metastasis, and drug resistance, particularly in the context of the safety of using EVs as regenerative therapies. This is particularly relevant because tissue repair after injury involves extensive stem cell activity; a myriad of remodeling and immunoregulatory factors (including on EVs) secreted by parenchymal, niche, stromal, and stem cells; and therefore an enhanced probability that a chronic wound can develop various grades of neoplasia (137). Considering that certain stem cells, especially ESCs or iPSCs, are particularly prone to formation of benign teratomas and malignant teratocarcinomas upon in vivo administration (95, 120), it would be interesting to determine if stem cell EVs play any roles in these processes. Because EVs are acellular, they are expected to be less tumorigenic than the intact stem cells from which they derive. Nevertheless, EVs, whether from stem cells, parenchymal cells, or neoplastic cells, have been reported to influence tumor formation, metastasis, and drug resistance (4, 28, 138–140). In particular, cancer stem cells (CSCs), a subpopulation of self-renewing cancer cells capable of giving rise to tumors (36, 137, 141–144), can depend on, or respond to, factors typically associated with embryonic development—in particular, Wnt, Hh, and Notch ligands discussed above, which are associated with membranes, including EV membranes. Thus, dysregulation of EVs carrying growth factors, morphogens, immune receptors, and certain nucleic acids might also be involved in induction, maintenance, or both of CSC activity (145), as well as in subsequent tumor growth and metastasis (138, 140, 146, 147), aspects that will be important to assess in future studies.

The literature is inconclusive as to whether stem cell EVs suppress or promote tumors. Part of the discrepancy may be due to the complexity of signals and pathways that are used by stem cells (e.g., MSCs) to sense and home to tumors and that may overlap with mechanisms that guide them to sites of injury and inflammation (123–125). Perhaps the pro- or antitumor properties of stem cells and their EVs depend on the conditions used to culture the stem cells and induce EV production and on the tumor model system used, as both the tumor microenvironment and the systemic environment of the host can vary greatly from tumor suppressive to tumor promoting (148–150). EVs from MSCs can transport tumor-supporting miRNA, proteins, and metabolites (151), and similarly, microvesicles from renal CSCs stimulate angiogenesis and formation of lung premetastatic niche (152). By contrast, a separate study reported that liver stem cell–derived microvesicles inhibited hepatoma growth by delivering antitumor miRNAs (153). In an early study, DC exosomes (sometimes referred to as dexosomes) eradicated tumors (154) via antigen presentation and costimulation immune mechanisms.

A recent, particularly illuminating study employed cre-lox techniques to provide evidence for EVs in mediating cancer metastasis (25). EVs from tumor cells expressing cre recombinase were taken up by proximal and distant tumor cells expressing a loxP-flanked STOP sequence in the gene encoding a fluorescent protein as a reporter, and the uptake of EV factors, such as certain mRNAs, correlated with enhanced metastasis of recipient, initially less malignant, tumor cells (marked by expression of fluorescent reporter protein). Likewise, lung cancer cells released EVs that induced phenotypic changes in recipient bone marrow cells (155). In addition, cancer cells have been reported to release EVs to induce anergy or apoptosis in immune cells (156), expel antitumor drugs via EVs (157), and eliminate targeting antibodies by shedding EVs carrying the target-antibody complexes (158), representing three novel mechanisms of tumor immune evasion and drug resistance.

In summary, although more data are needed, the above and related studies (Table 1) implicate dysregulated EVs as a new mechanism of neoplastic transformation, metastasis, and resistance (28, 138) and suggest caution is needed regarding the therapeutic use of stem cell EVs before their safety profiles, especially tumorigenesis, are validated thoroughly in vivo. By contrast, the link between EVs from stem cells or various cell types of the tumor microenvironment also suggests approaches to target cancer cells, including CSCs, selectively may be effective (36, 144) (e.g., by using EVs displaying particular Wnt or Hh factors to enhance their targeting, uptake, and processing). When targets in a particular tumor are known, EVs could also be engineered to deliver antitumor nucleic acids, membrane-bound antibodies, or small-molecule drugs. For example, MSC EVs were used to transfer anti-miR-9 to glioblastoma multiforme cells to reduce their chemoresistance by decreasing the expression of the drug efflux transporter P-glycoprotein (159).

## MANUFACTURING AND REGULATORY ASPECTS OF STEM CELL EVS

As with other biological agents in clinical use, EV products will require clinical good manufacturing practice (cGMP) protocols, including cell banking and storage, scalability, stability, lot and batch tracking, pathogen screening, and other physical, chemical, and biological methods for quality control (111, 160) (discussed below and in Table 2).

### Manufacturing EVs

**Figure 5:**
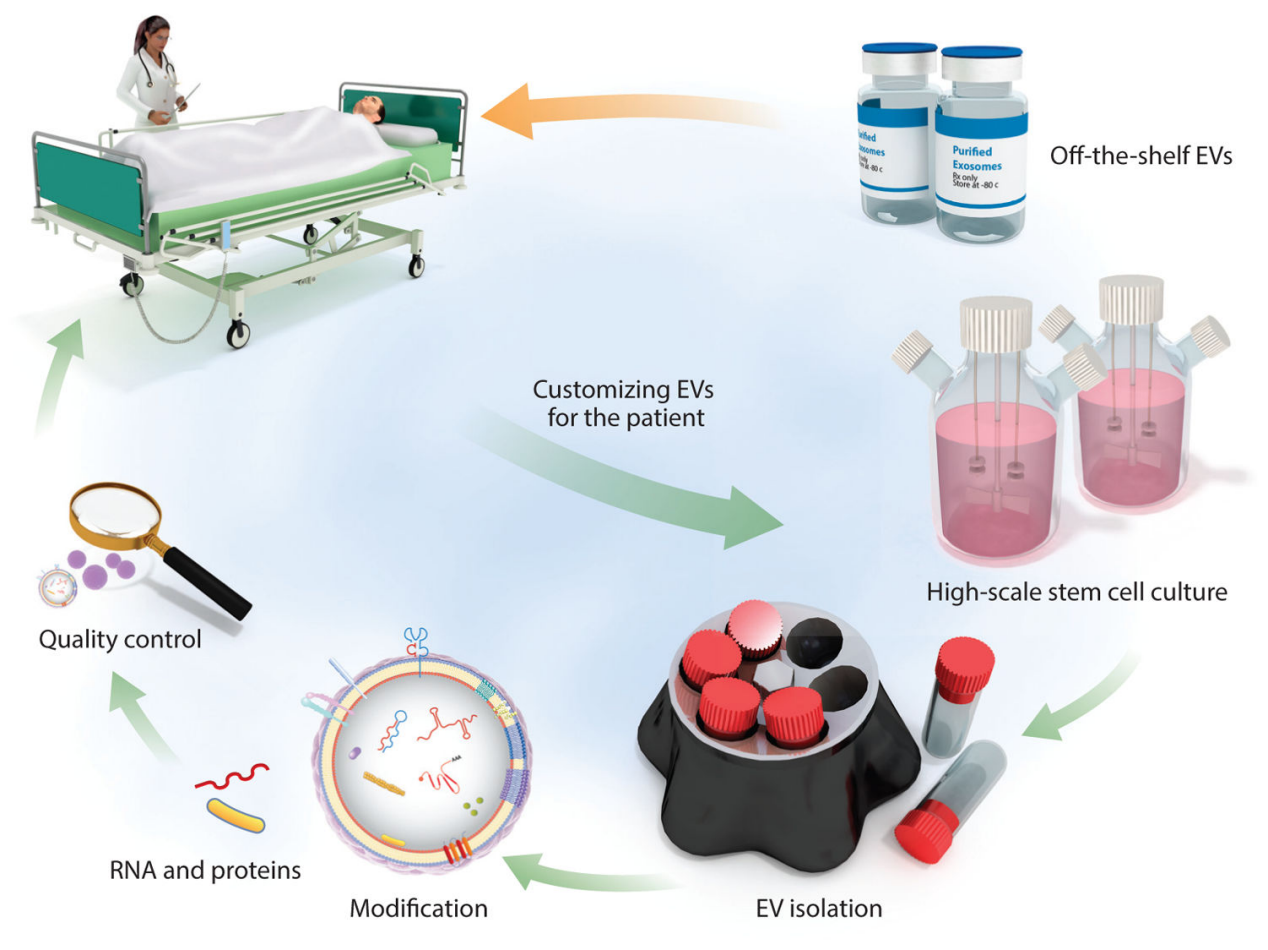
Stem cell extracellular vesicles (EVs) for clinical applications. EVs are designed, manufactured, and quality controlled beforehand and stored as off-the-shelf medications to be infused in patients as needed. Other EVs may also be customized accordingly for the individual patient by harvesting autologous stem cells, expanding and modifying them, producing EVs, and infusing them back to the same patient. In this process, the stem cells can also be genetically modified, and their EVs may undergo further modification by loading them with therapeutic molecules. Finally, EVs need to undergo quality control and be stored for future administration.

It is currently difficult to obtain sufficient quantities of EVs for large-scale in vivo animal studies and human clinical trials (111). In our experience, 1 L of MSC-conditioned media from a total of about 60 million MSCs yields about 1–2 mg (protein content) EVs, sufficient for experimentation in only a few mice. For comparison, depending on the disease model, EV therapeutic efficacy is approximately 50–500 μg (protein content) per administration in mice, whereas in human trials with stem cells, one typically administers 1 million whole cells/kg. EVs were administered to a GvHD patient using an escalating dose regimen beginning at a total EV-protein of 0.05–0.15 mg/kg and ending at 0.20–0.60 mg/kg (136). This first clinical study hints at future approaches to personalize treatment with EVs depending on the patient’s condition, whether using off-the-shelf EVs suitable for a variety of patient groups or deriving them from autologous stem cells expanded and modified ex vivo (Figure 5); in either circumstance, considerable quantities of EVs will be required.

One solution to the challenge of EV large-scale production would be to extract clinical-grade EVs from spent culture media (containing EVs) currently considered biotech waste (161). The current methods for isolating EVs are ultracentrifugation (current benchmark), polymer precipitation, size-exclusion chromatography (SEC), and TFF, but research continues to improve operational challenges and associated high costs. Both SEC and TFF have been used commercially for applications that include production of recombinant proteins and antibodies; such methods could therefore be modified for purification of EVs. Other approaches that could potentially meet the scale of EVs needed for clinical tests include cell fragmentation into membrane vesicles followed by loading with drugs (162) or nanoparticle envelopment with cell membranes (163, 164).

In addition to the difficulty in generating sufficient quantities of EVs that possess therapeutic efficacy, there are numerous safety considerations, which include toxicity and side effects, as well as the need for pharmacokinetic studies prior to approval for clinical use. The use of animal serum–free media is also recommended for EV products, as serum contains EVs that may influence recipient cell responses (165). As EVs possess a complex mixture of biological molecules, some of which may be countereffective, bioengineering tools may be used to avoid them. For example, stem cell lines could be engineered to lack class II MHC transactivator (*CIITA*) or MHC genes to reduce the potential immunogenicity of EVs and make them available for a genetically diverse group of patients.

### Regulatory and Quality Control Aspects of EVs

The size and complexities of EVs fall in the range between whole cells and defined single-molecule pharmaceuticals, similar to the clinically approved platelet lysate. Akin to cell-based and certain biological therapeutics, it is not possible to produce EVs under exact homogeneity from batch to batch as is done for chemically defined drugs. But this does not preclude their eventual adoption in clinical practice. Rather, just as occurs for therapy with whole cells (166), once the mechanism of action (MoA) has been delineated in full or in part, assays that test for the active ingredient or ingredients within specified parameters can assure EV product quality and potency. Thus, the quality control assays for EVs will likely combine features of cGMP testing of cells and the more traditional cGMP testing of chemical drugs (160, 167–169). Indeed, regulatory agencies classify EV preparations as biological medications, which are defined as one or more active substances derived from living cells. Regulatory frameworks for EV-based manufacturing and clinical trials exist in Europe, Australia, and the United States. EV-based therapies for human use in the United States are regulated by the Center for Biologics Evaluation and Research, a division within the Food and Drug Administration, and ISEV has recently published suggested guidelines on the use of EVs in clinical trials (170).

Similar to current cellular therapies in clinical trials (e.g., stem cells, immune cells) that can be tested for potency and other therapeutic parameters before administration in patients, stem cell–derived EVs can undergo potency and quality control testing (168, 169). The active substance in therapeutics determines their pharmaceutical classification and largely determines the MoA (167, 171). Where possible during early clinical development, defining the class or type of the active substance or substances responsible for the MoA in EVs will determine the pharmaceutical quality control strategy. Most of the reported active ingredients in EVs are largely in two classes: nucleic acids (especially mRNAs and miRNAs) and proteins (especially surface receptors and intravesicular enzymes or transcription factors) (Table 1). Once identified, active ingredients of EVs can be overexpressed as described in the preceding section to improve homogeneity and consistency of manufacturing. In fact, the investigation of EVs’ MoAs could lead to the discovery of single agents such as nucleic acids, peptides, or proteins that could be used therapeutically on their own, as has been done in the area of stem cell transplantation (11). However, if the specific molecule or molecules of the MoA have not been determined, a potency assay may be used to test for inactivation of the active ingredient (e.g., UV or gamma irradiation to inactivate nucleic acids, or proteases to remove surface proteins from EVs). In general, the in vitro potency assays will vary depending on EV type and therapeutic application (160). Homogeneity or consistency assays will likely also be needed to track selected biomarkers in each batch. Nonactive components in the final formulation of a drug (excipients) will also need to be characterized (171).

In conclusion, although for many therapeutic applications of EVs, the specific MoA might not be completely definable because of their complex nature, EVs are arguably more amenable than cells to logistical operations such as potency assays, freeze/thawing, batch tracking, and related quality control tests. As researchers continue to develop the technologies for analyzing EVs, the quality control for their characterization is also expected to develop further.

## STEM CELL EVS AS BIOMARKERS AND DIAGNOSTICS

EVs are being investigated increasingly as potential biomarkers of health and pathology (172–175), in what is termed exosome biopsy. There are several reasons for this: EVs are found in all bodily fluids (2) and can be accessed with relatively noninvasive protocols; EVs are released in a dynamic fashion and carry combinatorial signals; EVs host nucleic acids, whose analysis is particularly suitable using next-generation sequencing methods (176); and a growing number of preclinical studies of protein and nucleic acid composition of EVs (Table 1) may allow correlations with known biomarkers (177, 178). Most studies of EVs as biomarkers or EV-hosted biomarkers have been in cancer (179, 180), neurology (181–183), prenatal genetic testing (184, 185), immunology and hematology (40, 186, 187). The sidebar titled Selected Companies Conducting Research and Development on EVs lists some companies that develop exosome diagnostics.

SELECTED COMPANIES CONDUCTING RESEARCH AND DEVELOPMENT ON EVS **Aethlon** creates diagnostic tools to detect and quantify exosomes in blood and other fluids. Its subsidiary

**Exosome Sciences** is testing the Hemopurifier device in clinical trials to deplete cancer exosomes from patients.

**Anosys Inc.** (acquired by **Chromos Molecular Systems Inc.**) develops dendritic cell–derived EVs for new cancer therapies.

**Capricor Therapeutics Inc.** develops cardiac-derived stem cells and their EVs to repair damaged heart tissue.

**Caris Life Sciences** is a molecular informatics company with platforms for analysis of EVs from patients.

**Exosome Diagnostics** develops biofluid-based diagnostics to deliver personalized precision healthcare, particularly for cancer.

**Exovita Biosciences** develops exosome-based therapies to selectively target breast cancer cells.

**ReNeuron** uses neural stem cell–derived exosomes to treat neurological diseases.

**System Biosciences (SBI)** develops products for EV isolation, characterization, and labeling, including tools for next-generation nucleic acid analysis.

As cell-based medications become more widespread and enter clinical practice, researchers are developing newer methods to track the fate of the administered cells (such as the use of nucleic acid barcodes), similar to what is done in toxicology and pharmacokinetics and pharmacodynamics analysis of small-molecule drugs, antibodies, and other biologics. Similar to the analysis of endogenous EVs or cell-free nucleic acids in blood and other bodily fluids, EVs released by administered exogenous stem cells could be collected from body fluids and tested as potential biomarkers. In particular, the added versatility of introducing a marker (e.g., nucleic acid barcodes) in the injected cells allows researchers to monitor the fate of transplanted cells via their released EVs as a surrogate marker, which represents an exciting future direction. For instance, a few preclinical studies have tracked EV biodistribution and uptake using cre-lox reporter techniques (25, 26).

The properties of EV membranes that provide them with excellent biocompatibility and greater durability (38), and the ease of genetically engineering EVs (19) compared to liposomes and nanoparticles, make EVs potentially useful carriers for diagnostic and imaging agents. We believe this use will increase in the near future. Such probe-loaded EVs could enable the study of EV biodistribution and homing to target sites to both illuminate biology and detect diseases (e.g., tumors) in vivo (188). For example, conjugating a luciferase-encoding EV (189) with a cancer-specific antibody may provide such an in vivo diagnostic. EVs may also serve as in vitro diagnostics and biosensors, such as has been reported for EVs with G protein–coupled receptors (GPCRs) on chips to detect GPCR agonists or antagonists (190).

Overall, the properties of EVs, which derive from natural membranes and cytoplasmic material, have enabled their investigation for use as injury or disease biomarkers, potential surrogate markers for transplanted stem cells, or biosensing systems in various therapeutic and diagnostic models. Nevertheless, the utility of EVs as biomarkers of specific diseases and conditions is provisional, given the current paucity of studies in this emerging area and the difficulties and complexities of the biomarker field itself (178). Further studies are therefore needed on the diagnostic utility of EVs, especially to accompany their therapeutic roles.

## CONCLUSIONS AND PERSPECTIVES

EVs are emerging as mediators of stem cell responses during embryonic development and during adult tissue homeostasis and its restoration after injury. Parenchymal cells may emit EVs loaded with morphogens, apoptotic patterns, and immunomodulatory and other signals to activate their local stem cells or recruited bone marrow MSCs, which in turn can secrete EVs loaded with protein and nucleic acids interacting with specific pathways in recipient parenchymal cells to contribute to their healing or replacement. However, fundamental questions associated with the generation, distribution, and uptake of EVs should be addressed to fully understand the role and biology of stem cell EVs in cell-cell communication and tissue regeneration (see the sidebar titled Fundamental Open Questions in Stem Cell EV Biology and Biotechnology).

FUNDAMENTAL OPEN QUESTIONS IN STEM CELL EV BIOLOGY AND BIOTECHNOLOGY

- What are the mechanisms and implications of EV biogenesis?
- To what extent does the content of EVs mirror (or differ from) their originating cells?
- How are stem cell EVs loaded with specific membrane or intravesicular cargo?
- How are EVs targeted to and taken up by specific cells?
- What are the roles of EV size, stability, membrane curvature, and structural presentation and multivalency of membrane receptors in their biological and therapeutic effects?
- What role do EVs play for morphogen distribution and signaling during development?
- What is the significance of endogenous stem cell EVs in tissue repair?
- Do EVs produced by exogenous stem cells and administered in vivo exert therapeutic effects?
- What are the key components of stem cell EVs responsible for therapeutic efficacy?
- What are the best approaches for analyzing endogenous and exogenous EVs in vivo?
- Can stem cell EVs bypass biological barriers, and if so, what are the mechanisms?

Because EV biogenesis has evolved as an efficient type of cell-cell communication, EVs offer several advantages as therapeutics compared to cells, synthetic nanoparticles, and single molecules. As EVs are derived from cells and can be engineered to be nonimmunogenic, they can possess exceptional biocompatibility and biostability characteristics. Their small size enables EVs to avoid the pulmonary first-pass effect and to penetrate deep inside most tissues. For clinical applications, EVs offer additional advantages relative to whole cells, including lack of a nucleus to avoid neoplastic transformation, enhanced stability to freeze/thaw cycles, and greater capacity for loading with small molecules, proteins, and nucleic acids. Additionally, EVs can be engineered with specific receptors or antibodies on their surface to deliver therapeutic cargo into target cells and tissues. EVs may thus find increasing use as vehicles for gene therapy and drug delivery in medicine and as transfection reagents in biotechnology.

Booming EV research has sparked the emergence of numerous companies that aim to develop EV-based therapeutics or diagnostic biomarkers (see the sidebar titled Selected Companies Conducting Research and Development on EVs). The main current therapeutic applications of EVs are in stem cell–based therapy; autoimmunity and inflammation; heart, lung, and kidney diseases; neurodegenerative diseases; and cancer, although the list is expected to increase in the near future. Depending on patient subpopulations and applications, EVs can be banked in various genetic compatibility groups (e.g., depending on blood type, MHC haplotypes, or both) to be used as off-the-shelf medication (Figure 5). To further customize treatment depending on a patient’s condition, autologous stem cells can be expanded ex vivo, modified, and used to produce EVs, which would then be reinfused into the patient.

However, numerous challenges remain before stem cell EVs can be translated successfully into clinical use (Table 2). In particular, scale-up production is currently a bottleneck for sufficient quantities of clinical-grade EVs for humans, although this can be potentially addressed by feeding cGMP-grade cell culture supernatants into TFF operations.

In conclusion, the intercellular functions of EVs honed by evolution to maintain and restore homeostasis are being tested for personalized and regenerative medicine. Bioengineered stem cell EVs are therefore anticipated to find increasing applications in their niche between molecular and cellular medicine and may play a part in regenerative therapy by maturing together with other medical advances.

## Figures and Tables

**Table 1:** Select biomolecules on EVs from stem and parenchymal cells

Source cell(s) or tissue	EV biomolecule(s)	Recipient cell(s)	Reference(s)
ESCs	Proteins, mRNAs, miRNAs (various)	Fibroblasts	191
	Oct4, Rex1, Nanog, HoxB4, Sox2, miRNA290	HPCs, retinal Müller	115, 192
iPSCs	miRNAs (various)	Ischemic heart	193
HSCs	Tissue factor (CD142), mRNAs (various)	Subendothelial cells, blood cells (various)	194, 195
**MSCs**			
Umbilical cord	Wnt4	Burned skin	130
Bone marrow	mRNAs (various), Ang1, FGF7, miR-21, miR-34a, lncR-7SK, lncR-Y1	Kidney tubular, lung epithelial, endothelial, MCF7 cancer line	128, 131, 151
Adipose tissue	miRNAs (various), tRNAs (various)	NA	196
ESC-derived	Enzymes of oxidative phosphorylation	Ischemic heart	133
**Other tissue stem cells**			
Liver stem cells	mRNA (AGO2), miRNA (antiproliferative)	Hepatocytes, liver cancer	153, 197
Kidney cancer stem cells	miRNAs (angiogenic)	Lung endothelial	152
Endothelial progenitors	miR-126, miR-296	Pancreas endothelial	118
Cardiac stem cells	miR-146a	Cardiomyocytes, cardiac endothelial	119
Neural stem cells	IFNγ-IFNγR1	NIH 3T3	198
**Other cell types and tissues**			
Motor neurons, colon cancer, nodal cells, epidermal cells	Wg (Wnt), Evi (WLS)	Muscle, fibroblasts	56, 70, 71
*Drosophila* wing imaginal disk cells, chick notochord cells	Hh	NA (imaginal disk), anterior receiving cells (chick notochord)	75–77, 199, 200
Endothelial	Delta-like 4 (Notch ligand)	Endothelial	80, 81
Heart	AT1R	Cardiomyocytes, endothelial	201
Lung	mRNA for surfactant proteins B and C	Marrow	10, 88
Liver	miRNAs (various)	Hepatocytes	202
Kidney	Aquaporin 2	Kidney collecting duct cells	203
Brain	Neural proteins and nucleic acids (various)	Brain cells (various)	204, 205
**Immune system**			
PBMCs	miRNAs (various)	Blood cells (various)	206
DCs	MHCI, MHCII, antigenic peptides, costimulatory ligands	T cells	154, 207, 208
B cells	MHCI, MHCII, antigenic peptides, costimulatory ligands	T cells	209, 210
Jurkat T cell line	miRNAs (various)	APCs	211
Mast cells	mRNAs, miRNAs (various)	CD34^+^ HPCs, lung epithelial cell line	212, 213

Abbreviations: Ang1, angiopoietin 1; APC, antigen presenting cell; DC, dendritic cell; ESC, embryonic stem cell; EV, extracellular vesicle; FGF7, fibroblast growth factor 7; HPC, hematopoietic progenitor cell; HSC, hematopoietic stem cell; iPSC, induced pluripotent stem cell; MHCI, major histocompatibility complex class I; MHCII, MHC class II; MSC, mesenchymal stem cell; NA, not applicable; PBMC, peripheral blood mononuclear cell.

**Table 2:** Current challenges in translational research with stem cell EVs

Problems	Recommended solutions
Choosing the optimal stem cell type, culture, and stimulation conditions to produce EVs	Follow the profile of EVs from tissue-resident stem cells if known; fortify with RNA- or protein-targeting pathways of interest
Producing homogeneous EVs	Produce EVs within specified ranges of biomarkers and test for potency
Producing EVs on a large scale	Use bioreactors for culturing cells; use existing conditioned media to harvest EVs; force plasma membrane extrusion through filters to form EVs
Manufacturing clinical-grade EVs	Adapt existing cGMP production of cells and biologics for EVs
Characterizing clinical-grade EVs	Analyze protein and nucleic acid markers of EV batches; use lot and batch tracking
Using EVs as diagnostic reagents	Functionalize EVs with antibodies, quantum dots, and MRI reagents to label specific cells in the body or track the fate of injected cells; deposit bioengineered EVs on chips for diagnostics

Abbreviations: cGMP, clinical good manufacturing practice; EV, extracellular vesicle.

## SUMMARY POINTS

1. EVs are generated using evolutionarily conserved biomolecular mechanisms and function primarily in tissue-, organ-, and organism-wide intercellular communication to maintain or restore homeostasis.
2. EVs are carriers for the distribution of morphogens and growth and differentiation factors from tissue parenchymal cells to stem cells to inform on tissue health or disease state.
3. Stem cell–derived EVs carry proteins and nucleic acids that can support healing of injured tissues.
4. Various bioengineering approaches can use stem cell EVs as technology platforms for therapeutic and diagnostic applications.
5. For some regenerative therapies, natural and engineered EVs from stem cells may be superior to single-molecule drugs, biologics, whole cells, and synthetic liposome or nanoparticle formulations.
6. EVs may possess superior biocompatibility and biostability and pose fewer risks for abnormal differentiation or neoplastic transformation compared to whole cells.
7. Current challenges in the use of EVs as potential therapeutic alternatives to cells include scaling up cGMP manufacturing and improving associated quality control and batch tracking methods.
8. Future applications are expected to make increasing use of stem cell EVs as therapeutics, therapeutic carriers, and diagnostic or biopsy reagents.
